# Effects of fluoxetine and escitalopram on C-reactive protein in patients of depression

**DOI:** 10.4103/0976-500X.77091

**Published:** 2011

**Authors:** Nilesh Chavda, N. D. Kantharia, Jaykaran

**Affiliations:** *Department of Pharmacology, Government Medical College, Surat, India*

**Keywords:** Anti-inflammatory activity, C-reactive protein, erythrocyte sedimentation rate, Hamilton rating scale for depression, white blood cell count

## Abstract

**Objective:**

To study the anti-inflammatory activity of fluoxetine and escitalopram in newly diagnosed patients of depression and also to evaluate the association between depression and inflammation.

**Materials and Methods:**

Ninety-eight newly diagnosed patients of depression were recruited as cases. From these, 48 had started treatment with fluoxetine (20 mg/day) and 50 had started treatment with escitalopram (20 mg/day). After 2 months of treatment of these patients, Hamilton rating scale for depression (HRSD scale), C-reactive protein (CRP), erythrocyte sedimentation rate (ESR) and white blood cell (WBC) count were measured and compared to their respective baseline values before starting treatment. One hundred healthy volunteers were recruited as controls and their baseline of CRP, ESR and WBC count were measured and compared with their respective baseline values of cases. Severity of depression was measured by HRSD scale and anti-inflammatory activity was measured by reduction CRP, ESR and WBC count.

**Results:**

On baseline comparison between cases and controls, there were significant increases in the levels of CRP (*P* = 0.014), ESR (*P* = 0.023) and WBC count (*P* = 0.020) in cases. In fluoxetine (20 mg/day) treatment group, there was a significant reduction in the levels of CRP (*P* = 0.046), ESR (*P* = 0.043) and WBC count (*P* = 0.021) after 2 months of treatment but no significant reduction in HRSD scale (*P* = 0.190). Similarly, in escitalopram treatment group, there was a significant reduction in CRP (*P* = 0.041), ESR (*P* = 0.030) and WBC count (*P* = 0.017) after 2 months of treatment but no significant reduction in HRSD scale (*P* = 0.169).

**Conclusion:**

In newly diagnosed patients of depression, inflammatory markers such as CRP, ESR and WBC count were significantly raised and Selective serotonin reuptake inhibitors SSRIs such as fluoxetine and escitalopram reduced them independent of their antidepressant effect. So, SSRIs have some anti-inflammatory activity independent of their antidepressant action.

## INTRODUCTION

Selective serotonin reuptake inhibitor drugs are used in various psychiatric disorders. Some studies have shown that following SSRI treatment for major depression, there was a significant drop in C-reactive protein (CRP) concentrations, irrespective of whether or not the depression resolves. This shows that antidepressants induce an anti-inflammatory response independent of antidepressant action.[1] Similarly, in another study, sertraline therapy exerted immunomodulatory effects through a decrease in the proinflammatory cytokine interleukin (IL-12) and an increase in the anti-inflammatory cytokines IL-4 and tumor growth factor (TGF)-β1.[2] The drawbacks of these studies were small sample size, nonavailability of high sensitivity CRP assay and other confounding factors.[1,2]

Various studies have shown an association between depression and cardiovascular diseases.[3] High sensitivity CRP, which is considered as an independent risk factor for coronary artery disease and a marker for systemic inflammation, is reported to be increased in depression.[3,4] Depression is associated with immunological changes, and also, it is possible that depression may affect the development of coronary disease through systemic inflammation.[5]

So, this exploratory study was planned to see the effect of selective serotonin reuptake inhibitors such as fluoxetine and escitalopram on various inflammatory markers such as CRP, erythrocyte sedimentation rate (ESR) and white blood cell (WBC) count and also to evaluate an association between depression and inflammatory markers.

## MATERIALS AND METHODS

### Selection of subjects

#### Case group

Newly diagnosed patients of depression according to Diagnostic and Statistical Manual of Mental Disorders (DSM)-IV-TR criteria, who had recently started either fluoxetine (20 mg/day) or escitalopram (20 mg/day) treatment, formed the cases.

#### Control group

Healthy volunteers were recruited from blood donation camps.

The inclusion criteria for study group were:

1. Newly diagnosed patients of depression by DSM-IV-TR criteria;
2. Patients of either sex;
3. Within the age limit of 15–55 years; and
4. Patients who were willing to be enrolled for the study and who had given written informed consent.

The exclusion criteria for case group were the following.

1. Patients who were on any other medication that affects CRP, ESR and WBC count (anti-inflammatory drugs, oral contraceptive drugs, etc.).
2. Patients who were already taking any antidepressant medication.
3. Patients with any infection, e.g., bacterial/viral/fungal/mycobacterial.
4. Patients with any allergic complication of infection, such as rheumatic fever, erythema nodosum.
5. Patients with any inflammatory disease such as rheumatoid arthritis, juvenile chronic arthritis, ankylosing spondylitis, psoriatic arthritis, systemic vasculitis, polymyalgia rheumatica, Reiter’s disease, Crohn’s disease, familial Mediterranean fever, etc.
6. Patients with myocardial infarction, tumor embolization, acute pancreatitis.
7. Patients with trauma due to surgery, burns, fractures.
8. Patients having the habits of alcohol or/and smoking.
9. Patients with malignancies such as lymphoma, carcinoma, sarcoma.
10. Pregnant woman.

The inclusion criteria for control group were:

1. Healthy volunteer recruited from blood donation camp;
2. Persons of either sex;
3. Persons within the age limit of 15–55 years; and
4. Persons who were willing to be enrolled for the study and who had given the written informed consent.

The exclusion criteria of the control group were the same as the exclusion criteria for the study group.

### Experimental protocol

After getting permission from Institutional Ethics Committee, the investigator visited the Psychiatry OPD daily for screening of the patients and recruited 98 newly diagnosed patients of depression as cases who met with the inclusion and exclusion criteria. Depression was diagnosed using DSM-IV-TR criteria. Here, procedures of the study were explained to cases and written informed consent was obtained from all the patients. Out of these cases, 48 patients were randomized to fluoxetine (20 mg/day) and 50 patients were randomized to escitalopram (20 mg/day). Parameters such as high sensitivity CRP (hsCRP), ESR, WBC count and Hamilton rating scale for depression (HRSD scale) were measured at baseline and after 2 months of treatment in both (fluoxetine and escitalopram) the groups. To see the association between inflammation and depression, the investigator recruited 100 healthy volunteers as control group, as per the exclusion and inclusion criteria. Parameters such as CRP, ESR and WBC count were measured and compared with the case group. Here, CRP, ESR and WBC count were inflammatory markers and HRSD scale was showing severity of depression at that time. CRP was measured by immunoturbidometry method, ESR by Westergren method and WBC count using automatic cell counter. The protocol of the study was approved by Institutional Ethics Committee, and written informed consent was taken from all study participants (cases and controls).

### Statistical methods

Data obtained in our study were analyzed using the Statistical Package for Social Sciences (SPSS for Windows) software, version 16.

The distribution of the quantitative study data was checked by skewness, kurtosis, Komolgorov Smirnov test and Shapiro Wilk test. For evaluation of baseline variables, Chi-square test was used. Wilcoxon Signed Rank test was used to compare post-treatment HRSD scale of depression with pre-treatment HRSD scale (baseline) in fluoxetine and escitalopram treatment groups. Mann Whitney U test was used to compare HRSD scale of depression between fluoxetine and escitalopram treatment groups at baseline and after 2 months of treatment. Unpaired t test was applied to compare CRP, ESR and WBC count difference between case and control groups and also between fluoxetine and escitalopram treatment groups. Paired t test was used to compare CRP, ESR and WBC count difference between pre- and post-treatment of either fluoxetine or escitalopram group. Difference can be considered statistically significant when *P* < 0.05.

## RESULTS

Results showed there was significant increased level of inflammatory markers in cases as compared with controls [Table 1]. After 2 months of treatment with either fluoxetine or escitalopram, there was significant reduction in the levels of inflammatory markers in both the treatment groups. But there was no significant reduction in HRSD scale [Tables 2 and 3]. There was no statistically significant difference in baseline and after 2 months treatment parameters between fluoxetine and escitalopram treatment groups [Tables 4 and 5].

**Table 1:** Baseline comparison between case and control

Parameter	Case (n = 98) mean (SD)	Control (n = 100) mean (SD)	*P* value	95% CI of difference
CRP (mg/dl)	4.09 (2.58)	2.99 (3.53)	0.014	1.09 (0.23–1.96)
ESR (mm/hour)	35.73 (22.12)	26.87 (31.25)	0.023	8.86 (1.26–16.47)
WBC	9095.92 (3453.67)	8021 (2978.42)	0.020	1047.92 (171.61–1978.22)

Unpaired t test was used to detect differences in CRP, ESR and WBC count between case and control groups CI, confidence interval; CRP, C-reactive protein; ESR, erythrocyte sedimentation rate; WBC, white blood cell

**Table 2:** Effects of 2-month long fluoxetine (20 mg/day) therapy on HRSD and inflammatory parameters (CRP, ESR, WBC) in the patients of depression

Parameter	Baseline (n = 48)	After 2 months treatment with fluoxetine (n = 48)	*P* value	95% CI of difference
HRSD	25 (4.15)	25 (5.23)	0.190	
CRP (mg/dl)	4.04 (2.60)	3.92 (2.55)	0.046	0.15 (0.002–0.231)
ESR (mm/hour)	35.96 (22.41)	34.02 (20.76)	0.043	1.94 (0.07–3.81)
WBC	9143.75 (3486.01)	8347.92 (2798.25)	0.021	795.83 (125.74–1465.92)

Values of HRSD scale are in median (SD). Values of CRP, ESR and WBC are in mean (SD). Wilcoxon Signed Rank test was used to compare HRSD scale in two related samples. Paired t test was used to detect difference in CRP, ESR and WBC count between two paired groups, CI, confidence interval; CRP, C-reactive protein; ESR, erythrocyte sedimentation rate; HRSD, Hamilton rating scale for depression; WBC, white blood cell

**Table 3:** Effects of 2-month long escitalopram (20 mg/day) therapy on HRSD and inflammatory parameters (CRP, ESR, WBC) in the patients of depression

Parameter	Baseline (n = 48)	After 2 months treatment with escitalopram (n = 48)	*P* value	95% CI of difference
Scale	24.50 (4.67)	23 (5.92)	0.169	
CRP (mg/dl)	4.04 (2.59)	3.91 (2.52)	0.041	0.124 (0.005–0.244)
ESR (mm/hour)	35.96 (22.41)	33.88 (20.88)	0.030	2.08 (0.21–3.96)
WBC	9143.75 (3486.01)	8312.50 (2796.78)	0.017	831.25 (154.20–1508.29)

Values of HRSD scale are in median (SD). Values of CRP, ESR and WBC are in mean (SD). Wilcoxon Signed Rank test was used to detect the difference in the HRSD scale between two related groups. Paired t test was used to detect difference in CRP, ESR and WBC count between two related groups, CI, confidence interval; CRP, C-reactive protein; ESR, erythrocyte sedimentation rate; HRSD, Hamilton rating scale for depression; WBC, white blood cell

**Table 4:** Baseline comparison between fluoxetine and escitalopram groups before starting treatment

Parameter	Fluoxetine (n = 48) mean (SD)	Escitalopram (n = 50) mean (SD)	*P* value	95% CI of difference
HRSD	25 (4.15)	24 (4.78)	0.748	
CRP (mg/dl)	4.04 (2.60)	4.13 (2.59)	0.855	-0.096 (-1.14 to 0.94)
ESR (mm/hour)	35.96 (22.41)	35.52 (22.07)	0.922	0.44 (-8.48 to 9.36)
WBC	9143.75 (3486.01)	9050 (3457.10)	0.894	93.75 (-1298.62 to 1486.12)

Values of CRP, ESR and WBC are in mean (SD). Values of HRSD scale are in median (SD). Mann Whitney U test was used to detect the difference in the HRSD scale between two treatment groups. Unpaired t test was used to detect difference in CRP, ESR and WBC count between two treatment groups, CI, confidence interval; CRP, C-reactive protein; ESR, erythrocyte sedimentation rate; HRSD, Hamilton rating scale for depression; WBC, white blood cell

**Table 5:** Comparison of effects of 2-month long treatment of fluoxetine (20 mg/day) and escitalopram (20 mg/day) on HRDS and inflammatory parameters (hsCRP, ESR, WBC)

Parameter	Fluoxetine (n = 48) mean (SD)	Escitalopram (n = 48) mean (SD)	*P* value	95% CI of difference
Scale	25 (5.23)	23 (5.92)	0.481	
CRP (mg/dl)	3.93 (2.55)	3.91 (2.52)	0.975	0.016 (-1.01 to 1.04)
ESR (mm/hour)	34.02 (20.76)	33.88 (20.88)	0.957	0.23 (-8.20 to 8.65)
WBC	8347.92 (2798.25)	8312.5 (2796.78)	0.951	35.42 (-1098.40 to 1169.23)

Values of CRP, ESR and WBC are in mean (SD). Values of HRSD scale are in median (SD). Mann Whitney U test was used to detect the difference in the HRSD scale between two treatment groups. Unpaired t test was used to detect difference in CRP, ESR and WBC count between two treatment groups, CI, confidence interval; CRP, C-reactive protein; hsCRP, high sensitivity C-reactive protein; ESR, erythrocyte sedimentation rate; HRSD, Hamilton rating scale for depression; WBC, white blood cell

## DISCUSSION

In our study, we found significant increase in inflammatory markers in newly diagnosed patients of depression as compared to healthy controls. Several studies had previously examined the association between CRP and depression, and the preponderance of evidence supports the conclusion that depression is associated with increase in CRP but was confounded or mediated by other variables. There were various factors such as body mass index (BMI), age, lipids, gender, exercise, alcohol, smoking, various medical conditions, use of medications, physical injury, or medical procedures, and all these factors associated with elevated CRP.[6] From these confounders, we tried to exclude most of them still our results might be confounded by BMI and level of lipids.

Some earlier studies have shown the probable mechanism of CRP rise in depression. Depression might promote an inflammatory response by activating the immune response.[7] Alternatively, the effects of depression on inflammation might be due to its links to psychological stress. The latter has also been associated with excessive production of IL-6.[8,9] IL-6 and IL-1β synergistically induce a systemic immune response. IL-6 is also the main proinflammatory cytokine inducing synthesis of type 1 acute phase proteins such as CRP. Also, psychological stress has been shown to increase oxidative state, which in turn, through modified lipids and lipoproteins, is thought to initiate an inflammatory response in the artery wall.[10,11] It may also be that depression leads to higher CRP levels, but it does so through some indirect, nonbiological mechanism such as a health behavior. One possible explanation for rise in CRP is that in individuals with high levels of stress, stress activated pathways contribute to increases in CRP. It is recognized that depressive symptoms are associated with enhanced stress-induced norepinephrine responses and norepinephrine dysregulation.[12] Preliminary evidence suggests that norepinephrine-dependent adrenergic stimulation results in activation of the nuclear factor (NF)-KB, a transcription factor known to increase gene expression for cytokines, such as IL-1β and TNF-α, and chemokines, such as IL-8, monocyte chemoattractant protein-1(MCP-1) and macrophage inflammatory protein(MIP-1a). Similarly, NF-KB activation also promotes IL-6 gene expression.[13] Severity of depressive symptoms, anger, and hostility, alone and in combination, are associated with increased gene expression of proinflammatory cytokines and chemokines[14–16] and elevated plasma levels of IL-6.[17] Thus, individuals who show elevated levels of stress may respond to daily life stressors with excessive stress-induced sympathetic activation that triggers an NF-KB–dependent cascade of proinflammatory events that contribute to increases in CRP.

Our results showed reduction in inflammatory markers after 2 months of treatment with either fluoxetine or escitalopram. Various previous studies supported our findings. In one study, 20 depressed female patients were treated for 3 weeks with either fluoxetine (20 mg), paroxetine (20 mg) or sertraline (50 mg), and when CRP concentration was measured following 3 weeks of antidepressant treatment, levels of this protein had significantly reduced. This finding was independent of the response to antidepressant therapy.[1] Sluzewska *et al*, (1996) measured CRP in 49 in-patients with major depression, all of whom had been drug-free for at least 10 days. A statistically significant difference in CRP concentration was found in the patients.[18] Rothermundt *et al*, (2001) also found that CRP levels following 2 and 4 weeks of treatment did not differ significantly from those in healthy participants, providing further evidence for the anti-inflammatory potential of antidepressants.[19] CRP level was measured following 3 weeks of antidepressant treatment and they found that levels of this protein had significantly reduced.[20] Similarly, in another study, sertraline therapy exerted immunomodulatory effects through decrease in the proinflammatory cytokine IL-12 and an increase in the anti-inflammatory cytokines IL-4 and TGF-β1.[2] This finding was independent of the response to antidepressant therapy checked by HRSD. This study had a limitation of small sample size and hsCRP assay would have yielded more accurate results.

The reduction in inflammatory markers may be because of SSRI reducing proinflammatory cytokines and increasing anti-inflammatory cytokines.[21] This fall in cytokines leads to fall in inflammatory marker and inflammation.

In our study, we found no significant reduction in HRSD scales after 2 months of treatment with either fluoxetine or escitalopram. In contrast to the previous reviews,[22,23] now considerably more evidence supports a preeminent role for central nervous system (CNS) dopaminergic (DA) circuits,[24] with many investigators suggesting that the now well-documented suboptimal therapeutic responses to SSRIs and selective serotonin–norepinephrine reuptake inhibitors (SNRIs) may be due, in part, to their relative lack of effect on brain DA circuits. In addition to the very impressive evidence of reduced activity of serotonergic neurons in depression as assessed in postmortem, cerebrospinal fluid (CSF), and neuroendocrine studies, there are new data from both postmortem and positron emission tomography (PET) imaging studies demonstrating a reduction in the number of serotonin transporter (SERT) binding sites (the site of action of SSRIs) in the midbrain and amygdale of drug-free depressed patients, as well as a reduction in both presynaptic (in the midbrain) and postsynaptic (in the mesiotemporal cortex) 5HT1A (5-hydroxytryptamine, 5-HT) receptor density. Taken together, these data suggest a net reduction in the number and/or function of the presynaptic 5HT nerve terminals and a reduction in postsynaptic serotonergic signal transduction, at least at one of the 5HT receptor subtypes. Previous studies demonstrated an increase in 5HT2 receptor density, perhaps due to a relative decrease in 5HT availability. These may be the reasons for there being no improvement in depression scale.[25,26]

There are some limitations in our study. Confounding factors such as BMI, lipid, etc. associated with elevated CRP may affect the results. Cause–effect relationship between depression and CRP was not established. IL-6 may induce production of corticotropin-releasing hormone, resulting in hypercortisolemia,[27] which in turn might contribute to depression. The latter might also represent a vicious cycle ultimately leading to an exacerbation of depression, immunosuppression, and inflammation. An ideal analysis would be the assessment of change in depressive scores and CRP over time, and their correlation and interaction as they relate to cardiovascular events. Also, here we failed to establish dose–effect relationship of SSRIs. Control group should be followed up for comparison. Large sample size could give better results.

In our study, we used hsCRP, ESR and WBC count as markers of inflammation, which were not used in previous studies.

## CONCLUSION

From our study, we conclude that inflammatory markers were raised in newly diagnosed patients of depression as compared to controls, and 2 months of treatment with either fluoxetine or escitalopram significantly reduced the inflammatory markers, independent of their antidepressant effect. So, these SSRIs have some anti-inflammatory actions independent of their antidepressant action.

Future studies can be planned with selective serotonin receptor agonist, NF-KB antagonist and other drugs to explore the mechanism of anti-inflammatory effects. A definitive way to test this concept would be the use of novel drugs that specifically block CRP binding and its proinflammatory effects *in vivo*,[28] which can be a powerful tool for determining whether increased CRP production merely reflects atherosclerosis or does indeed participate in its pathogenesis and complications, and they could also have cardioprotective effects in depressed patients.
